# Biocorona on Iron Oxide Nanoparticles in a Complex Biotechnological Environment: Analysis of Proteins, Lipids, and Carbohydrates

**DOI:** 10.1002/smsc.202300064

**Published:** 2023-07-09

**Authors:** Lucía Abarca-Cabrera, Olga Milinovic, Viktoria Heitler, Broder Rühmann, Jürgen Kudermann, Massimo Kube, Hendrik Dietz, Volker Sieber, Sonja Berensmeier, Paula Fraga-García

**Affiliations:** ^1^ School of Engineering and Design Department of Energy and Process Engineering Chair of Bioseparation Engineering Technical University of Munich (TUM) Boltzmannstraße 15 85748 Garching Germany; ^2^ Chemistry of Biogenic Resources Technical University of Munich (TUM) Campus Straubing Schulgasse 16 94315 Straubing Germany; ^3^ Catalysis Research Center (CRC) Technical University of Munich (TUM) Ernst-Otto-Fischer-Straße 1 85748 Garching Germany; ^4^ Department of Biosciences School of Natural Sciences Technical University of Munich (TUM) Am Coulombwall 4a 85748 Garching Germany; ^5^ Munich Institute of Biomedical Engineering Technical University of Munich (TUM) Boltzmannstraße 11 85748 Garching Germany

**Keywords:** biomolecules, bio–nano interactions, bionanotechnology, bioseparation, magnetic nanoparticles

## Abstract

Upon their introduction into a biological environment, nanoparticles are spontaneously covered by a variety of biomolecules, forming a (multi)layer called the “biocorona”. However, the interaction of small and large molecules with nanosized materials is not fully understood and in complex aqueous systems, even less, limiting their exploitation. The objective is to gain insights into the mass partitioning between the solid and the liquid phases for the most abundant groups of biological molecules in a biotechnological milieu. Herein, the biocorona composition is analyzed after the exposure of bare iron oxide nanoparticles to *Microchloropsis salina* lysates to evaluate the influence of the environment's pH, temperature, and ionic strength on the adsorption of proteins, lipids, and carbohydrates. Maximum adsorption capacities reach at pH 4.0 and yield 0.47, 0.08, and 0.11 g g^−1^ for proteins, fatty acids, and carbohydrates, respectively. The increase in ionic strength and temperature of the environment promotes protein adsorption, the decrease in temperature raises fatty acid adsorption, and acidic pHs foster the adsorption of the three types of biomolecules. Abundance of the biomolecules plays a key role in the biocorona content. This approach should lead to further studies on complex systems to modulate the adsorption at the bio–nano interface.

## Graphical abstract

This study reports the quantification of proteins, lipids, and carbohydrates found in microalgae that adsorb onto bare iron oxide nanoparticles. A massive accumulation of these biomolecules from the multicomponent system can be achieved on the surface. The ionic strength, temperature, and pH of the liquid phase affect the resulting adsorption capacities, depending on the types of biomolecule.

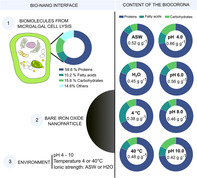

## 1 Introduction

Magnetic nanoparticles have been gaining attention for research in the Life Sciences in the past three decades, mainly due to their magnetic moment, which lowers operating costs and accelerates processing, for example, when they are used in magnetic separation.^[^ ^1^ ^]^ One of the properties of magnetic nanoparticles, more precisely bare iron oxide nanoparticles (BIONs), is when added into a biological solution, different types of molecules are attracted to the nanoparticle surface, forming a halo called the “biocorona”.^[^ ^2^ ^]^ Hence, a crucial challenge in exploiting BIONs is to regulate the interactions between biological materials and nanomaterials at the bio–nano interface,^[^ ^3^ ^]^ specially for downstream processing applications, where one or more target molecules must be separated with high yield and purity from complex environments, such as cell lysates.

Magnetic materials have already been employed to isolate different types of biomolecules. Some attempts have been made to obtain the desired selectivity using surface decoration with designed ligands,^[^ ^4^ ^]^ which requires complex syntheses, thus raising the cost. Therefore, the surface modifications of nanomaterials make them too expensive for a broader range of applications, primarily when the final targets are not high‐value products. Some studies reveal that obtaining selectivity in bare nanomaterials is feasible.^[^ ^5^ ^]^ Therefore, an in‐depth understanding of the interactions and influences between different macromolecules and BIONs could fill the gap in regulating the adsorption profile of different classes of biomolecules and enable the use of these inexpensive materials as a bioseparation tool.

Numerous studies have focused on the bio–nano interactions, typically for proteins,^[^ ^6^ ^]^ given the interest in biomedical applications and the well‐established methods to analyze them, resulting in much research developing around the concept of the “protein corona”.^[^ ^7^ ^]^ Despite their critical role in biological systems and being nearly as abundant as proteins, the other bio(macro)molecules, that is, lipids, sugars, and nucleic acids, have received less attention regarding their interactions with nanomaterials, even though they are known to adsorb onto nanoparticles like proteins do.^[^ ^8^ ^]^ A possible reason is that studying and quantifying these molecular groups is more complex with the currently available analytical methods. Lipids and polysaccharides have a vast mosaic of chemical compositions, structural organizations in aqueous systems, and molecular weights: for polysaccharides, many branching degrees, and for lipids, multiple structures.^[^ ^9^ ^]^ There are only limited number of studies addressing the interaction of other classes of biomolecules with nanoparticles in complex environments. Physiological fluids are yet the preferred solutions studied, including the analysis of cholesterol, triglycerides and phospholipids,^[^ ^10^ ^]^ a native pulmonary surfactant,^[^ ^11^ ^]^ glycans,^[^ ^12^ ^]^ and circulating cell‐free DNA.^[^ ^13^ ^]^ Other scenarios start to be considered, including food matrices in which carbohydrates such as fructose, glucose, sucrose, and maltose in honey and fatty acids from olive oil are studied;^[^ ^14^ ^]^ plants, analyzing total carbohydrates adsorbed from xylem fluids;^[^ ^15^ ^]^ or aquatic ecosystems, where metabolites are investigated.^[^ ^16^ ^]^

A first step toward a thorough understanding of the biomolecule–nanoparticle interplay is then to broaden the scope of the studies to other complex environments beyond the physiological one and to analyze different classes of biomolecules. This is necessary to gain insights into cooperativity and competitiveness effects in real biological systems. We have recently demonstrated the significance of such interplays using a model system with a mixture composed of three classes of biomolecules.^[^ ^17^ ^]^ In the present work, we quantify the three main classes of biomolecules (based on their abundance in the cell mass) in a biologically complex mixture, that is, from the cell lysates of the microalga *Microchloropsis salina* (*M. salina*), adsorbed onto BIONs. Microalgae are a useful biomaterial source for different applications, from fuels to high‐value products. In particular, *M. salina* is a promising marine microalga that has been successfully cultivated at a large scale and produces long‐chain polyunsaturated fatty acids.^[^ ^18^ ^]^ Biomolecule concentration and separation using BIONs is a promising tool for obtaining cost‐effective products from microalgal biomass feedstock to increase the profitability of the process. Most of these compounds of interest are stored intracellularly, necessitating cell disruption and, as a consequence, all other components are released into the medium and must be separated in subsequent steps.^[^ ^19^ ^]^

We further explore the effect of the environmental conditions of the liquid phase on the accumulation of biomolecules on the inorganic surface by varying the environment's ionic strength, pH, and temperature. We then characterize the surface employing zeta potential, dynamic light scattering (DLS), and Fourier‐transform infrared (FTIR). The most important contributions are the data on the evolution of the adsorbed biomolecule quantities related to the nanoparticle–biomaterial mass ratio as well as the presentation of adsorption data for a very large number of individual polysaccharides and fatty acids.

The present work broadens our knowledge and understanding of bio–nano interactions with valuable data on the three main classes of biomolecules in complex nonphysiological environments. The investigation here is a step toward developing strategies to control the adsorption of different types of biomolecules in nonphysiological complex systems with regard to the formation of corona after exposure to the multicomponent system *M. salina* lysate with BIONs. The final goal is to control adsorption with a selective partition of molecules between the liquid and the solid phase to fully use the biomass to obtain various high‐value coproducts through biorefinery.

## 2 Results and Discussion

### 2.1 Description of the System

The bio–nano interface comprises three main entities: the nanoparticle surface, the surrounding milieu, and the biomolecules. In order to understand the mechanisms that shape the interactions between these elements, the main characteristics of each element must be identified.

The first element consists of BIONs. The BIONs used in this study have an average diameter of 11.7 nm, according to transmission electron microscopy (TEM) measurements, and 7.6 ± 0.6 nm from X‐ray diffraction (XRD), with a surface area of 96.9 ± 0.2 m^2^ g^−1^. For the full characterization, see Abarca‐Cabrera et al. (2022).^[^ ^17^ ^]^

The second element, the milieu, is artificial seawater (ASW),^[^ ^18^ ^]^ a hypersaline solution of dissolved mineral salts (Table S1, Supporting Information) with a molarity of 0.5 m, a conductivity of ≈45 mS cm^−1^, and a pH of 8.0.

**Figure 1:**
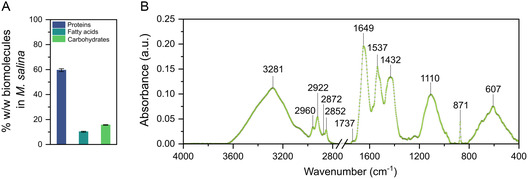
Characterization of *M. salina* lysate obtained from cell disruption by bead milling. A) Mass composition of total proteins, fatty acids, and carbohydrates in *M. salina* lysates (relative to the total biomass, i.e., without the salt content). An experiment of *n* = 3 is represented. Error bars correspond to the minimum and maximum values. B) FTIR of the cell lysate at a concentration of 1 g L^−1^ in ASW.

The third element is the microalgal biomolecule from *M. salina*. Microalgae are photosynthetic microorganisms found in freshwater and marine environments, which are considered a source of many beneficial bioactive compounds, such as proteins (5–74% w/w), lipids (7–65% w/w), carbohydrates (8–69% w/w), and other metabolites including pigments and vitamins (1–14% w/w).^[^ ^20^ ^]^ In the *M. salina* lysates examined, the total protein, fatty acids, and carbohydrates were 59.6%, 10.2%, and 15.6%, respectively (**Figure** **1**A). We further analyzed the FTIR spectra to identify the peaks related to the main biomolecules and to use these peaks as a reference to detect possible changes in the chemical bonds after the formation of the biocorona (Figure 1B). For proteins, two amide groups of protein chains were identified at 1649 cm^−1^ for amide I and 1537 cm^−1^ for amide II.^[21a]^ Lipid bands from the hydrocarbons, such as fatty acids, are in the region 3050–2800 cm^−1^: 2960 and 2922 cm^−1^ for the ν_as_(CH_3_) and ν_as_(CH_2_) asymmetric stretches and 2872 and 2852 cm^−1^ for the ν_s_(CH_3_) and ν_s_(CH_2_) symmetric stretches, respectively.^[^ ^21^ ^]^ The ν(C=O) esters of triacylglycerols (TAGs), neutral lipids, are at 1737 cm^−1^.^[21a]^ ν(C–O–C) stretches of carbohydrates are observed in the region 1200–1000 cm^−1^.^[^ ^21^ ^]^

### 2.2 Biocorona: Proteins, Lipids, and Carbohydrates Meet an Inorganic Surface

We further investigated whether the BIONs adsorb the three major classes of biomolecules: proteins, lipids, and carbohydrates. Although these classes of biomolecules in interaction with nanoparticles have been investigated for some systems,^10^, ^12^, ^22^ to the best of our knowledge, this is the first study that analyzes a complex microbial‐derived environment, including the study of the three biomolecule groups altogether, and presents the mass‐related evolution of the adsorbed layer depending on different original concentration profiles for the solid adsorbent and for the biomass.

**Figure 2:**
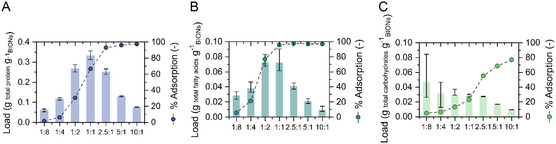
Adsorption loadings of total proteins A), total fatty acids B), and total carbohydrates C) from microalgal lysates for different BION‐to‐biomass mass ratios (left axis). The adsorbed percentages are shown for each point (right axis). The *M. salina* biomass concentration was kept constant, while the BIONs concentration was varied to achieve the desired nanoparticle‐to‐biomass ratio. Mean of three experiments is represented. Error bars represent the minimum and maximum values.

The biocorona content was first quantified for total proteins, total carbohydrates, and total fatty acids (**Figure** **2**). The three main classes of biomolecules adsorb onto BIONs: due to the tendency of nanoparticles to minimize their surface energy, they attract biological molecules.^[^ ^3^ ^]^

The ratio of particles to biomass was varied from 0.125 to 10 to determine the influence of the surface area available in the loading capacities and to elucidate whether the concentration profile of the solid support and the molecules modifies partitioning. Note that complete adsorption of proteins and lipids appears achievable, whereas, for polysaccharides, incredibly high particle concentrations are necessary to adsorb most of them onto the solid support. Moreover, the increase in adsorbed lipids is steeper than for the proteins: at the mass ratio 1:1, all the fatty acids are separated, while only 60% of the proteins are adsorbed and about 20% of the sugars. However, the total masses accumulated for each type of molecule reveal a substantially higher protein mass adsorbed for each nanoparticle‐to‐biomass mass ratio, which is unsurprising given that there are a lot more proteins than fatty acids in the lysate. For the ratio 10:1, where far more BION mass is in the system than biomass, low loadings are obtained, forming incomplete biocorona layers as the molecules are distributed across the entire available surface area. We expected that through the decrease in BION concentration, the surface might saturate, establishing the maximum load limit of the biocorona on the inorganic material and that the biocorona would stop growing at some point. Nevertheless, surprisingly, for proteins and fatty acids, a maximum load is achieved when the nanoparticle mass and the biomass in the system are similar (1:1 ratio for proteins and between 1:1 and 1:2 ratio for fatty acids), but the load decreases when the nanoparticle‐to‐biomass ratio decreases. A possible explanation is that more biomolecule–biomolecule interactions take place when more biomass is in the system than inorganic mass. As the BION's surface area decreases, the proteins interact with each other, forming agglomerates or aggregates.^[^ ^23^ ^]^ Moreover, in the case of lipids, the molecules are known to organize themselves into solution after a threshold concentration, forming structures such as micelles or vesicles.^[^ ^24^ ^]^ As opposed to the other two classes of biomolecules, the BIONs load of carbohydrates (mass of carbohydrates per unit mass of BIONs) continuously increases inversely to the nanoparticle‐to‐biomass ratio (as seen from right to left in Figure 2C): the more nanoparticles are in the system, the less carbohydrates are separated per unit mass of BIONs. This increase might be associated with the fact that this class of biomolecules has low‐affinity interaction sites for self‐association and in general, molecular interactions where carbohydrates are involved are commonly classified as weak interactions.^[^ ^25^ ^]^ The weak interactions with a low number of strongly charged functional groups might also explain the lower degree of interaction with the solid nanoparticles in the presence of proteins (carboxyl and amino groups) and fatty acids (carboxyl groups). Low adsorption capacities have been reported for carbohydrates in iron oxide and clay minerals surfaces, with values from 0.001 to 0.2 g g^−1^.^22^, ^26^ In our experiments, even in the presence of 10 times more nanoparticle masses than biomass masses, the whole carbohydrate content is still not recovered: only up to 77% is adsorbed. However, in the presence of high quantities of BIONs, the BIONs strongly agglomerate, probably decreasing the surface area exposed for interaction and adsorption (Figure S1, Supporting Information). Nonetheless, at this stage, we still do not know how much biomass is able to accumulate between individual nanoparticles. A biomass halo appears to surround the BIONs for lower nanoparticle‐to‐biomass ratios, but the resolution in our TEM pictures is not enough to confirm this effect. We think that adsorbing almost all proteins (up to 97%) and almost all lipids (up to 98%) from a solution and concentrating them on the nanoparticle surface is already an achievement and could have a significant impact in some life science fields. Moreover, we speculate that the reason for the higher tendency of carbohydrates to remain in solution could be related to the fact that generally, sugars possess less strongly charged functional groups, and therefore, they are less prone to electrostatic interactions in the presence of large quantities of proteins and lipids as competitors for the surface binding sites.

Previously, Roloff (1965) reported that one ton of montmorillonite, a clay mineral, can accumulate up to 550 kg proteins, 150 kg fatty acids, and 200 kg carbohydrates.^[^ ^27^ ^]^ However, this data on biomass accumulation did not originate from an experimental setup in the lab over a short time through simultaneous adsorption onto the solids. The accumulation formed in soil layers (*strata*), and the total mass quantities were obtained after very long time periods. In our study, one gram of dry nanoparticles can separate a maximum of 0.33 g proteins, 0.072 g fatty acids, and 0.05 g carbohydrates in competitive adsorption; these are the same dimensions as the ones in the old soil deposits. These results raise some essential questions: is there a maximum amount of biomass that can accumulate on inorganic minerals? Or, as long as we add more BIONs, will it be possible to separate all molecules in a solution? What is the role of the metal ions in solution on the adsorption behavior?

While the relation between the accumulated proteins‐to‐lipids and proteins‐to‐carbohydrates is quite similar to Roloff's ratios, in our study, the total accumulated masses are lower. The BIONs have accumulated less biomass compared to the soil reserves, considering that the magnetite density should be far higher than that of, for example, montmorillonite (approx. double).^[^ ^28^ ^]^ These results lead to further questions: would more time lead to an increase in the amount of biomass adsorbed? Would a higher biomass‐to‐BIONs ratio lead to a similar amount adsorbed for the different molecule types, taking into account the density differences between both minerals? In the future, we plan to determine if more biomass accumulates over time to see if there is a maximum biomass that can be adsorbed per surface area of mineral material and if this total accumulated biomass is independent of the surface of the mineral material. At the moment, our data and the knowledge we have gained from varied adsorption experiments with nanoparticles seem to point to a monolayer distribution. It also raises the possibility that for higher biomass concentrations, the system's free energy is lower after covering the surface with a biocorona, and the biomolecules remaining in solution prefer to organize themselves and/or agglomerate rather than to increase the number of layers on the surface. As soon as the pristine nanoparticle surface is biologically passivated, adding further biomolecules to the surface does not seem necessary. The maximum loadings of total proteins obtained in our experiments corroborate values obtained in other systems, varying from 0.2 to 0.4 g g^−1^.^[^ ^29^ ^]^ Nevertheless, for fatty acids, the maximum capacity was quite low in comparison with other adsorption studies with pure solutions,^[^ ^17^ ^]^ even though the initial concentrations of the fatty acids were similar in the microalgae, meaning values around 0.7 g L^−1^. This points to a difference in the competition situation compared with that of the single molecule. For carbohydrates, as reported, the loadings are, in general, very low in iron oxide surfaces.^22^, ^26^

It is important to add that at this stage, we have not examined the effect of the total suspended concentration present in the system, that is, when higher concentrations of solids and biomolecules are present for the same BION mass‐to‐biomass ratios. The higher the total concentrations of suspended condensed matter in the system (i.e., including the biomass and the nanoparticles), the more the van der Waals forces will be responsible for the agglomeration of nanoparticles and the agglomeration/precipitation of the biomolecules on the solid support. Furthermore, the effect of magnetic forces will also increase, possibly further increasing the total biomass separation ratios.

### 2.3 Biocorona Content at Different Physicochemical Conditions

#### 2.3.1 Protein Corona

Proteins are the most studied biomolecular class in terms of corona formation around nanoparticles; in the literature, the data are discussed in relation to nanoparticle characteristics (size, shape, charge, or material), the influence of incubation time, initial concentrations, and protein profile, among other conditions investigated.^7^, ^30^ In this study, we performed sodium dodecyl sulfate polyacrylamide gel electrophoresis (SDS‐PAGE) to identify the proteins adsorbed onto the BIONs. The main bands are presented for the sizes: ≈15, 25, 26, 55 kDa, and in the range of 26–43 kDa. Ribulose‐1,5‐biphosphate carboxylase oxygenase is an enzyme involved in carbon fixation, localized in the chloroplasts,^[^ ^31^ ^]^ and has been observed in different microalgae lysates.^[^ ^32^ ^]^ This enzyme is said to be one of the most abundant on Earth.^[^ ^33^ ^]^ With a size of 540 kDa, this enzyme consists of 8 small subunits of 14 kDa and 8 large subunits of 56 kDa.^[^ ^34^ ^]^ Due to the denaturing conditions used for the SDS‐PAGE, we hypothesize that the subunits of RuBisCO are separated, and the bands at 15 kDa constitute the small subunit, while the bands at 55 kDa correspond to the large subunits. As the rest of the proteins are in the range of the light‐harvesting complexes reported in the literature, that is, from 25 to 45 kDa,^[^ ^35^ ^]^ we speculate that the bands of 25, 26, and from 26 to 43 kDa form part of this antenna complex.

**Figure 3:**
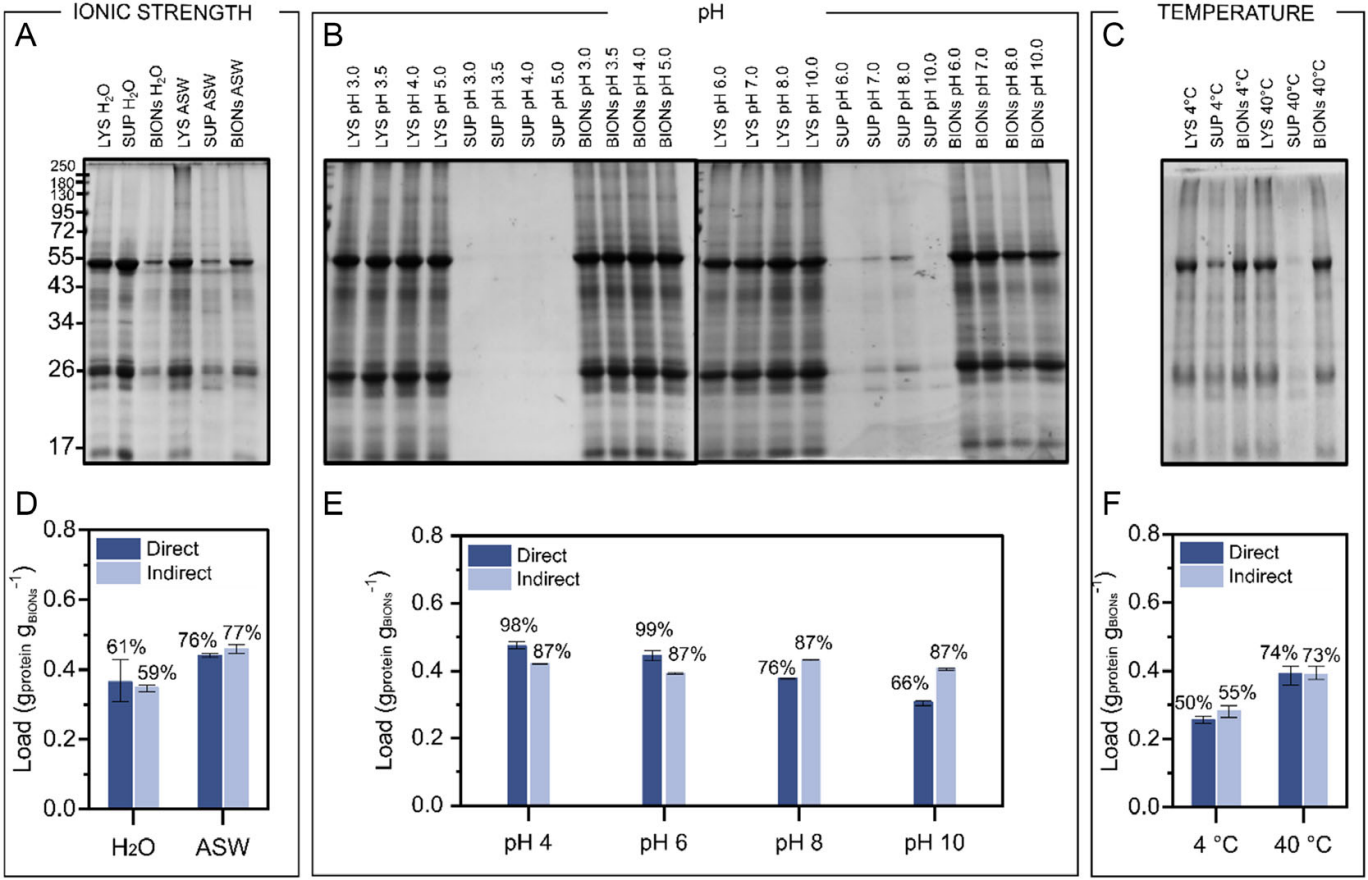
A–C) SDS‐PAGE of proteins in the lysate (LYS), in the supernatant (SUP), and onto the BIONs (BIONs) obtained after contact with microalgal lysates and BIONs at a 1:1 BIONs‐to‐biomass masses ratio in the presence and absence of salts from the ASW media (A), at pHs ranging from 3.0 to 10.0 (B), and at temperatures of 4 and 40 °C (C). D–F) Quantification of total adsorbed protein by direct calculation of proteins on the solids (dark blue) and by indirect calculation from the measurement of the protein content in the supernatant (light blue) in the presence and absence of salts from the ASW media (D), at pHs ranging from 4.0 to 10.0 (E), and at temperatures of 4 and 40 °C (F). Mean and the minimum and maximum values of three independent experiments are represented. LYS: lysate. SUP: supernatant recovered after the magnetic separation. BIONs: solids after magnetic separation. “Direct” represents the values measured directly from the BIONs, while “indirect” represents the values calculated from the analysis of the concentration remaining in the supernatants.

We studied the adsorption of the proteins for a variety of environmental incubation conditions, ensuring all of them are relevant for realistic situations achievable using typical processing steps in biotechnological productions. To evaluate the influence of salt ions, temperature, and pH, we varied these parameters in order to establish if specific conditions help certain biomolecules to adsorb better in comparison to others and to identify if there are any differences in the behavior of biomolecule classes in relation to the quantity adsorbed (**Figure** **3**).

For ionic strength, we compared two simple conditions which should provide information about the impact of the salt content on the biomass accumulated as a corona. The situation in which the cells were lysed in their own growth media (conductivity of ≈45 mS cm^−1^), which corresponds to the ASW, was compared with the situation where the cells were washed out through repeated centrifugation, supernatant discharge, and resuspension in deionized water, and the cells were then lysed in deionized water; the latter procedure enabled us to remove most of the salts (conductivity of 0.4 mS cm^−1^). As expected, the salt enhances the total adsorption capacity for proteins (Figure 3D). The salting‐out effect due to an increase in ionic strength decreases protein solubility, leading to the precipitation of proteins on the inorganic surfaces. Moreover, according to the Derjaguin, Landau, Verwey and Overbeek theory (DLVO theory), for high ionic strength, van der Waals forces (solely attractive forces) gain dominance over electrostatic forces and may attract the biomass to the surface.

pH is a factor that contributes to determining the charge of proteins^[^ 17, 36 ^]^ and of the nanoparticle surface.^[^ ^17^ ^]^ Figure 3B shows that electrostatic interactions play an important role in the adsorption of proteins. At pHs from 3.0 to 10.0, the lysate is mainly negatively charged (Figure S3, Supporting Information), while the nanoparticle surface is positively charged below its isoelectric point (IEP), which is 7.1.^[^ ^17^ ^]^ As soon as the nanoparticle becomes negatively charged (occurring after the IEP), bands of proteins in the supernatant appear, indicating that the proteins are being electrostatically rejected. This repulsion also occurs at pH 8.0 but not at 10.0. At this latter pH, we presume that the multivalent ions from the media increase the protein adsorption as previously observed^[^ ^37^ ^]^ due to crystal nucleation of these multivalent ions, calcium, and magnesium, that form positively charged precipitates that accumulate on the BIONs’ surface and increase the binding capacities of proteins due to electrostatic attractions. The effect of pH is stronger for protein adsorption without the presence of salts (Figure S2, Supporting Information), probably because then the water ions only interact with the proteins, and no competing proton association or dissociation reactions of salt ions take place. With regard to the effect of temperature on the adsorption profile, higher adsorption is obtained at higher temperatures, which might be related to the increase in the solubility of the proteins or the decrease in the viscosity of the liquid phase, factors that may enhance the diffusion of the molecules (Figure 3F). The mixtures, where the temperature effect was tested, are around pH 8.0 and conductivity of 40 mScm^−1^ (Table S2, Supporting Information).

In general, the adsorption capacities achieved are similar to other studies where monolayer distribution on the nanoparticle surface was calculated,^[^ ^29^ ^]^ which leads to the assumption that proteins, even in complex systems, form a monolayer and completely distribute onto the total solid surface. Moreover, such adsorption capacities were corroborated through a direct mass balance using the total protein measured in the BIONs and an indirect mass balance using the supernatants, which verified the same behavior under the conditions studied (Figure 3).

#### 2.3.2 Lipid Corona

Lipids have rarely been studied in terms of biocorona formation.^[7b]^ Cholesterol, triglycerides, lipoproteins, and bilayer membranes, all in physiological fluids,^10^, ^30^, ^38^ and total lipids in plant tissues^[^ ^39^ ^]^ have been studied.

**Figure 4:**
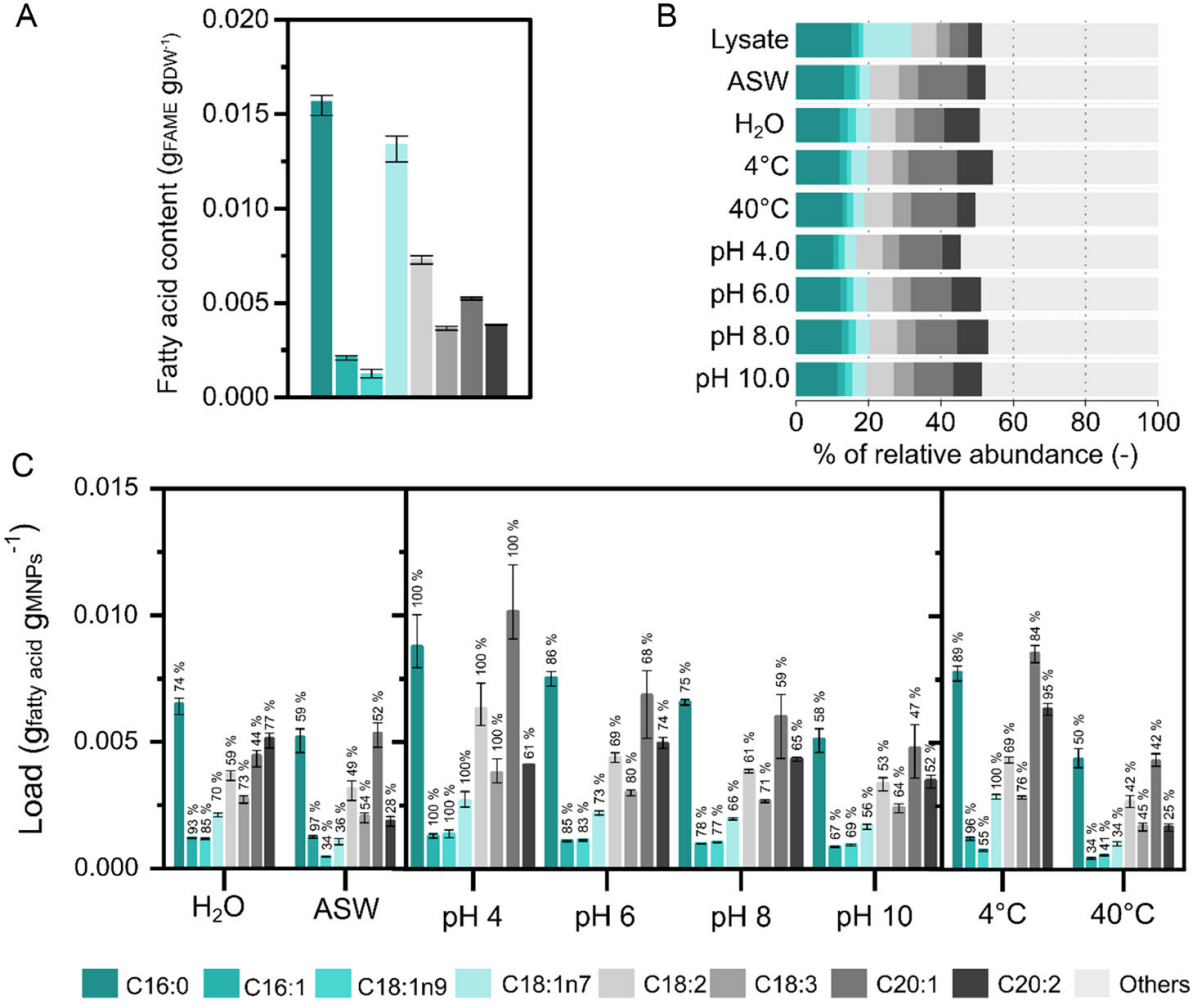
Quantification of fatty acid methyl esters (FAMEs) from microalgal lysates adsorbed onto BIONs in 1 h at 1000 rpm and 25 °C at a ratio 1:1 BIONs‐to‐biomass. A) Fatty acid content of *M. salina* lysates under native conditions, pH ≈8.0; the biomass dry weight (DW) is determined after washing twice and salt removal. B) Relative abundance of fatty acids in the lysate and in the biocoronas (content adsorbed onto the solids) formed in different environmental conditions. C) Individual loading and the percentage of adsorption (related to the total of each fatty acid) of the most abundant fatty acids from *M. salina l*ysates in different environmental conditions. Fatty acids: C16:0 (palmitic acid), C16:1 (palmitoleic acid), C18:1n9 (oleic acid), C18:1n7 (vaccenic acid), C18:2 (linoleic acid), C18:3 (linolenic acid), C20:1 (eicosenoic acid) and C20:2 (eicosadienoic acid). Experiments were carried out in technical triplicates. Error bars represent the minimum and maximum values.

Microalgae produce a large number of lipid‐like compounds. Fatty acids are one of the most abundant lipids, constituting between 5% and 60% of cell dry weight.^[^ ^40^ ^]^ They are amphiphilic molecules with a polar head group (a carboxylic acid) and an aliphatic tail, which can be saturated (SFA, saturated fatty acids) or unsaturated (MUFA, monounsaturated fatty acids and PUFA, polyunsaturated fatty acids). Microalgae can produce fatty acids with chain lengths of up to 24 carbon atoms and can be present in unsaturated, monounsaturated, or polyunsaturated forms.^[^ ^40^ ^]^ Here, the fatty acid content of the *M. salina* lysates was analyzed using gas chromatography (GC). For the fatty acids, the quantities obtained are similar to other microalgal systems.^[^ ^41^ ^]^ **Figure** **4**A shows the masses of the eight most abundant fatty acids in the microalgae lysate; these represent about 50% of the total fatty acid mass in the lysate (Figure 4B).

In order to investigate the fatty acids assembled on the biocorona in nonphysiological environments under the same conditions used to analyze protein adsorption, the fatty acid content adsorbed was quantified to identify which fatty acids were retained on the surface and if their behavior showed notable differences to each other. Figure 4C shows the loading of the eight most abundant fatty acids in the different proposed environments.

To evaluate the influence of the ionic strength on the fatty acids’ adsorption, we analyzed the fatty acids in ASW media and deionized water (without salts). As observed in Figure 4C, there are no significant differences between either condition, although there are slightly higher loadings in the system without salts, which might be related to the fact that the high salt content in ASW leads to a shielding and consequently, a weakening of electrostatic interactions. Besides the minimal influence of ionic strength on the adsorption of fatty acids, we also evaluated the effect of different pHs on the adsorption of fatty acids. As with proteins, the highest adsorption of the total fatty acids occurs at pH 4.0, and the loadings steadily decrease from pH 4.0 to 10.0. The potential explanation for this behavior is that as the cell wall of the microalgae is negatively charged (Figure S3, Supporting Information), and the surface of BIONs is positively charged at pH 4.0, most of the organic material is probably adsorbed by electrostatic interactions, though without any preference for fatty acids. This phenomenon also occurs when a high surface area is available, as can be seen for 10:1 BIONs‐to‐biomass ratios (Section 2.2). Temperature, another parameter evaluated, affects the adsorption capacities of fatty acids on BIONs. At 4 °C (the lowest temperature studied), the decrease in temperature of the solution increases the pKa values,^[^ ^42^ ^]^ leading to a higher concentration of undissociated (protonated) fatty acids species; therefore, we expect less electrostatic repulsions at the native pH of the system (pH ≈8.0) and hence, higher adsorption capacities. The maximum adsorption capacity for total fatty acids was achieved at pH 4.0, obtaining 0.08 g g^−1^ (Table S4, Supporting Information). At this condition, almost all the eight individual fatty acids analyzed were adsorbed completely.

As the main purpose of this study is to identify potential conditions to achieve selectivity (differences in the adsorption capacity of particular types of molecules), three criteria are discussed to highlight the importance in the adsorption of fatty acids: initial concentration in the system, pKa, and hydrophobicity.

Although it is not possible to control the concentration profile of the biomolecules involved in complex environments, their abundance plays a key role in the composition of the biocorona. The large amount of a specific molecule in a mixture ensures a prevalence in the biocorona due to the increase in collision rate of that type of molecule with the surface and therefore increased probability of interaction. Protein adsorption studies using physiological media have demonstrated that albumin, the most abundant protein in these systems, tends to be one of the most adsorbed proteins^7^, ^43^ due to the mere probability of encountering the inorganic surface. In our system, palmitic acid (C16:0) is the most prominent fatty acid in the lysate (Figure 4A). This fatty acid was not adsorbed completely at the BION‐to‐biomass mass ratio used. However, it still has a high relative abundance onto the solid surface with respect to the other fatty acids due to the initial abundance (Figure 4B), similar to the situation of albumin from physiological environments in the biocorona. In mesoporous silica‐based nanoparticles, palmitic acid was completely recovered from a simulated microalgal oil.^[^ ^44^ ^]^ For proteins in human plasma, the abundance of each protein in the biocorona is not exactly in the same order as in the initial solution,^[^ ^12^ ^]^ as in our system for fatty acids. However, high concentrations of the biomolecules in the initial solution would determine their presence in the biocorona. The concentration of biomolecules in the solution is of special importance for lipids, which creates structures above a certain concentration called critical micelle concentration (CMC). The CMCs of all fatty acids studied here are higher than 1 mM,^[^ ^24^ ^]^ but the concentrations in the lysates are <1 mm (Table S5, Supporting Information). Therefore, the formation of structures such as micelles is not expected; however, the structural organization also depends on the presence of other fatty acids, (bio)molecules and salts. At the moment, we do not know if this might influence the behavior of the studied fatty acids in solution.

pKa, which measures the degree of ionization, plays a very important role in the adsorption of fatty acids, as the IEP does for proteins. If the pH of the solution is less than the pKa, the fatty acids are mainly in their protonated form; if the pH is greater than the pKa, they are more deprotonated. pKa values increase with fatty chain length and decrease when the number of double bonds increases.^[^ ^45^ ^]^ Considering this principle, the order of the fatty acids analyzed based on their pKa value is: C20:1 > C18:1 > C16:0 > C20:2 > C18:2 > C18:3 > C16:1 (Table S3, Supporting Information). In previous studies with sodium oleate,^[^ ^17^ ^]^ we observed that when this sodium salt of oleic acid is in its protonated state, the overall charge is still negative but lower than when it is in its deprotonated form. In this system, eicosenoic acid (C20:1) is the fourth‐most abundant in the lysate. However, in the loading profile, it occupies first place at pH 4.0 and second in all other pHs, competing in proportion with palmitic acid. The complete adsorption of C20:1 at pH 4.0 might be due to the fact that its pKa is the farthest from the pH of the solution. Therefore, it maintains its protonated state over a wider range of pHs, avoiding electrostatic repulsions when the nanoparticle surface switches to a negative charge. In this context, the same argumentation can be applied for palmitoleic acid (C16:1), as its pKa is the closest to the pH of the solution, and its loadings are the lowest of all the fatty acids studied in the complete pH range studied.

The hydrophobicity of the fatty acids depends on their structure: the longer the alkyl chain and the more unsaturated these fatty acids are, the higher their hydrophobicity. Moreover, the branching in the structure decreases the hydrophobicity.^[^ ^46^ ^]^ Therefore, the order of the fatty acids analyzed based on their hydrophobicity is as follows: C20:2 > C20:1 > C18:3 > C18:2 > C18:1 > C16:1 > C16:0. We expected that those fatty acids with higher hydrophobicity might adsorb in greater proportions onto solid surfaces than those with lower hydrophobicity due to their tendency to leave the solution. However, hydrophobicity does not appear to promote the selective adsorption of a particular fatty acid, as no increase in the adsorption percentages is observed as hydrophobicity increases. This might also be due to the fact that the total quantity of fatty acids is not high enough to show this expected hydrophobicity effect.

Our data demonstrate differences in the fatty acid partitioning between the solid and the liquid phase as an interplay between the abundance of each fatty acid and its particular physicochemical characteristics, such as its pKa. However, the value of pKa and abundance are not parameters that totally explain the capacity differences, and clearly, there are more characteristics that play a role. For vaccenic acid (C18:1n7), neither abundance nor pKa promotes high quantities of it on the biocorona.

#### 2.3.3 Carbohydrate Corona

Carbohydrates are a type of biomacromolecule whose role in biocorona formation has received almost no attention.^[^ 12, 47 ^]^ Their importance is not only as the main source of energy and as a structural component of living organisms but also for their association with other molecules to form glycoproteins or glycolipids. For microalgae, polysaccharides are mainly found in the form of starch grains, glycolipids (intracellularly), as cell wall constituents, and exopolysaccharides (EPS).^[^ ^48^ ^]^

**Figure 5:**
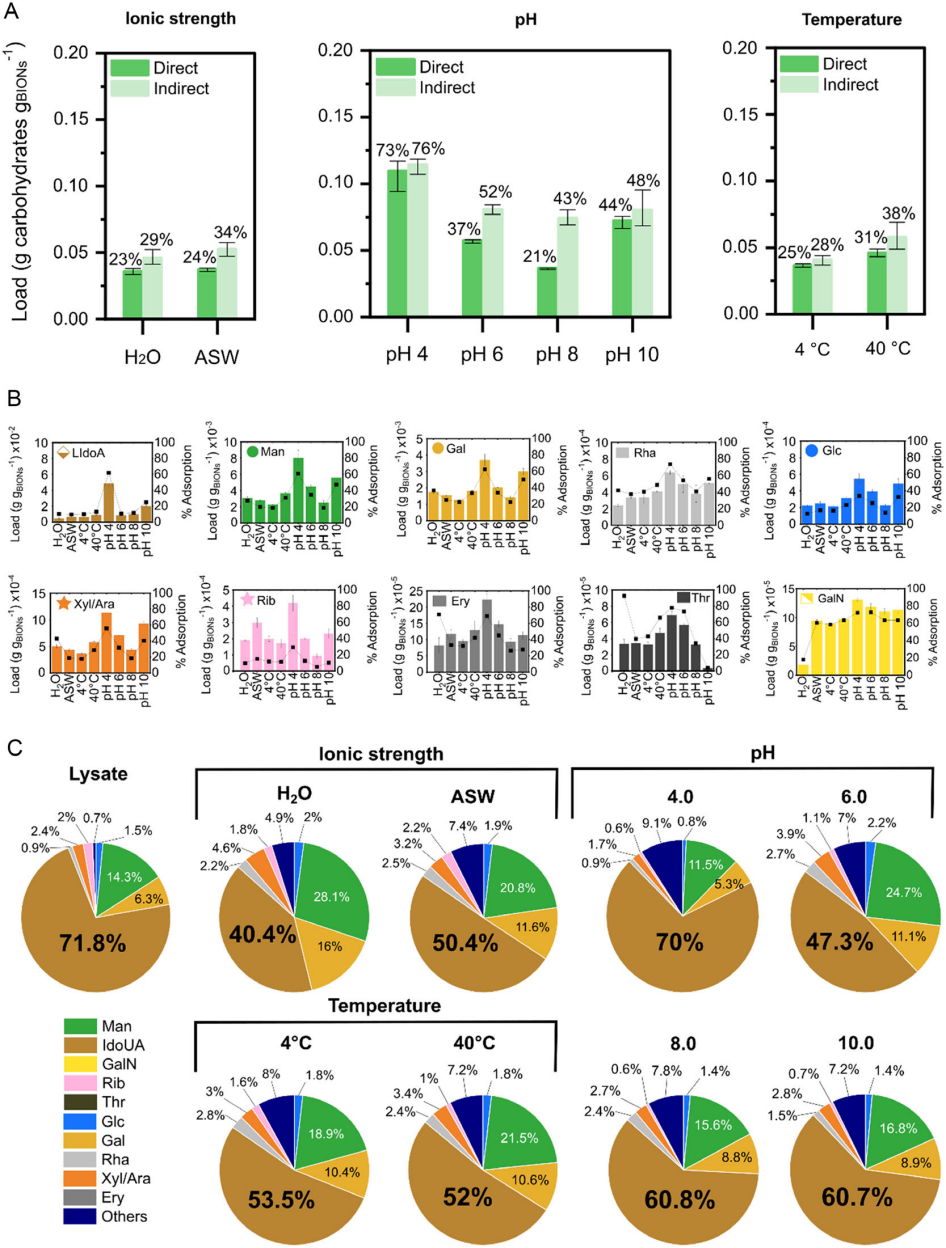
A) Loading of total polysaccharides using glucose as standard onto BIONs after 1 h incubation at 1000 rpm and 25 °C under native conditions of the lysate. The data include the comparison of values through direct measurement on the solids (dark green) and by indirect calculation from the measurement of the carbohydrate content in solution (light green). Mean of *n* = 3 independent experiments are represented. Error bars represent the minimum and maximum values. B) Adsorption mass of the most abundant monosaccharides onto BIONs; masses obtained by acid hydrolysis of the carbohydrates onto BIONs after incubation with microalgal lysates using a ratio 1:1 for the given environmental conditions and subsequent magnetic separation. The mean value is included with error bars representing the minimum and maximum values. C) Relative abundance of monosaccharides from microalgal lysates adsorbed onto BIONs at different environmental conditions at a ratio of 1:1 BIONs‐to‐biomass.

Carbohydrates generally adsorb to a lesser extent than proteins and fatty acids,^[^ 15, 22, 26 ^]^ not only in reference to the total adsorbed mass but also to the relative number of molecules on the solid surface compared to the aqueous environment. In order to investigate the effect of environmental conditions on the adsorption of microalgal carbohydrates onto BIONs, phenol‐sulfuric acid was used to quantify total carbohydrates in the cell lysates and on the BIONs after adsorption. Three different parameters were varied during the adsorption, as previously done with proteins and fatty acids: ionic strength, pH, and temperature (**Figure** **5**).

NaCl is known to break the hydrogen bonds of galactomannan, increase the flocculation or precipitation of chitosan, and change the process of gelatinization of carrageenan.^[^ ^49^ ^]^ Consequently, similar physicochemical changes of some carbohydrates were expected to influence the adsorption capacities, but as Figure 5A shows, the presence of salts from the ASW media, including NaCl (the most abundant one), does not appear to improve or reduce the loading of total carbohydrates.

Although the effect of salts on carbohydrate adsorption is not commonly discussed in terms of biocorona formation, temperature has already been evaluated as a parameter in other systems containing sugars. Lactose adsorbs to ZnO nanoparticles, reaching adsorption capacities ranging from 8 to 13% of the lactose in skimmed milk and from 29 to 37% when only lactose was in the solution. However, no significant effect was shown in the adsorption capacities of lactose at different incubation temperatures.^[^ ^50^ ^]^ On the other hand, fructose and glucose from honey incubated with SiO_2_ nanoparticles adsorb in a concentration and temperature‐dependent manner. The general pattern is that as temperature increases, higher loadings are achieved.^[^ ^14^ ^]^ Although there are discrepancies in the literature, in our system, the loadings appear to increase slightly at higher temperatures (Figure 5A), which may be attributed to a decrease in the viscosity of the medium and an increase in the diffusion of the molecules.

pH strongly affects the adsorption of carbohydrates, increasing the capacities at acidic conditions close to the percentage of separated proteins and lipids, as previously reported.^[26a]^ The maximum adsorption was 73% at pH 4.0, which is comparable to those systems in which 10 times more BIONs are used at the native pH of the mixture (i.e., 8.0) (Figure 2C and 5A). We speculate that the adsorption of particular polysaccharides increases the adsorption of total carbohydrates, especially those such as EPS that are negatively charged.^[^ ^51^ ^]^ Microalgae are recognized as a source of EPS^[^ ^52^ ^]^ and other charged polysaccharides, such as chitosan or carrageenan, that adsorb onto oppositely charged surfaces via electrostatic interactions.^[^ ^53^ ^]^ Another potential explanation is that, as the cell wall is negatively charged and polysaccharides are components of this structural layer, they adsorb without any selectivity but as a whole entity with proteins and lipids onto BIONs which are positively charged at acidic pHs.

As the final goal of our work is to determine whether selective adsorption can be attained, we analyzed the individual loading of the monosaccharides adsorbed onto BIONs using high‐performance liquid chromatography (HPLC) coupled with mass spectrometry (MS) after acidic hydrolysis and 1‐phenyl‐3‐methyl‐5‐pyrazolone high‐throughput method (HT‐PMP) derivatization. Due to the complexity of current methods to quantify the glycomic content in complex matrices, we did not analyze the intact polysaccharides.

Carbohydrate abundance in the biocorona is directly linked with its original abundance in the solution. This pattern has been observed in food environments, where sugars from honey are adsorbed accordingly at their initial concentrations,^[^ ^14^ ^]^ and in human plasma, where sialic acid, one of the most abundant monosaccharides in humans, enriches the biocorona.^[^ ^12^ ^]^ Unexpectedly, the most concentrated monosaccharide found in the cell microalgal lysates was iduronic acid (IdoA), the major uronic acid component of the glycosaminoglycans (GAGs), such as heparin, while typically, glucose would be the most abundant monosaccharide in microalgae due to the presence of starch and cellulose (glucose‐based polymers). The absence of glucose could be related to the fact that the microalgae were used after storage at 4 °C in the dark and not immediately after harvesting, resulting in the consumption of these carbohydrates by the microorganism. In the biocorona formed in our system, the five most abundant monosaccharides, namely IdoA, Man, Gal, and Xyl/Ara, are also the most abundant sugars in the microalgal lysate, together representing 86–89% of the glycome content (Figure 5C), corroborating our previous statement in the lipid section that the original abundance of a molecule plays an important role for its appearance in the biocorona, at least in the time frames of our incubation experiments. Remarkably, the sixth most abundant monosaccharide should be ribose (Rib); however, rhamnose (Rha) was in sixth place in abundance in all the biocoronas formed under the different conditions. Rib represents only 2% of the total carbohydrate content, while Rha is only 0.9%; hence, the concentrations are quite similar, and there are other factors that determine the priority to adsorb. Rib has a simple linear form, while Rha has a methyl group, which may give it a higher binding affinity, as observed for metabolites in anionic silica nanomaterials.^[^ ^54^ ^]^ Although IdoA is the most abundant carbohydrate in the biocorona, it does not completely adsorb (Figure 5B). In fact, the percentages of adsorption are below those of the other major components, such as mannose (Man) and galactose (Gal). Uronic acids, such as IdoUA, are part of the anionic nature of the EPS.^[^ ^55^ ^]^ This charge produces higher adsorptions at pH 4.0, where the BIONs have positively charged surfaces, but at other pHs, the electrostatic repulsions lead to lower loadings for this monosaccharide. In general, the adsorption of carbohydrates is concentration dependent, and low adsorption values are achieved. As we had observed previously for dextran,^[^ ^17^ ^]^ the environmental conditions under which we tested seem not to impact the total polysaccharides abundance on the corona as strongly as for lipids and proteins (Figure 5A). However, at pH 4, there is a clear increase in the adsorption capacity of all analyzed individual monosaccharides; this can be translated into higher adsorption of all polysaccharides at the lowest tested pHs, which might be enhanced by electrostatic interactions due to the positive charge of the nanoparticles at low pHs. Note that the values through direct measurement on the BIONs and by calculation from the measurement of the carbohydrate content in the supernatant for pH 6.0 and 8.0 are distant between each other, that could be related that at these pHs; the sugars are less strongly attracted by electrostatic interactions as the BIONs are around their IEP, and during the washing steps, more sugars are lost.

### 2.4 Further Characterization of Biocorona

**Figure 6:**
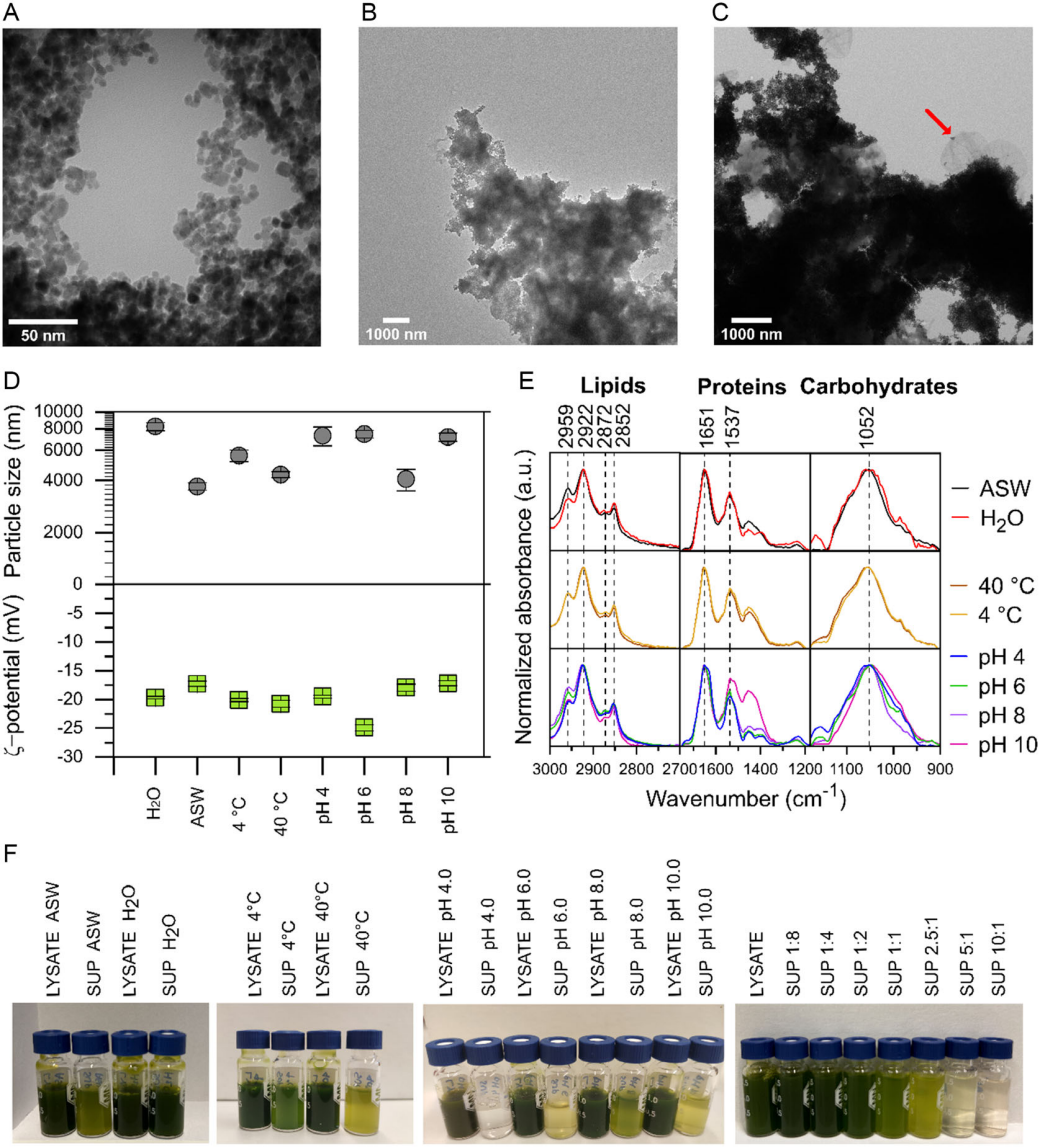
Surface characterization of BIONs after adsorption with microalgal lysate. A–C) TEM images of BIONs after exposure to lysates in deionized water (A,B) and at pH 4.0 in ASW (C) at a biomass‐to‐nanoparticle ratio of 1:1. D) Zeta potential and hydrodynamic diameter by DLS. The bars represent mean of *n* = 3 independent experiments and error bars show the minimum and maximum values. E) FTIR spectra of BIONs after exposure for 1 h to lysate under native conditions, normalized with the maximum peak of each quadrant. F) Photographs of lysates and supernatants after separation of BIONs exposed to the lysates.

The biocorona is a complex coating that provides a new biological identity to the nanoparticles’ surface. Therefore, the new characteristics obtained after the exposure of the BIONs to the microalgal lysate were investigated to identify the changes to the biocorona resulting from the different conditions tested (**Figure** **6**).

TEM images show BIONs after exposure to microalgal lysates. Figure 6A shows the morphology of the BIONs; however, the biocorona thickness cannot be measured as it is not clearly observed. A cloud‐like layer is observed on top of the BIONs (Figure 6B), which we assume is the biomass adsorbed, but the biocorona as a halo surrounding the surface cannot be seen in these complex systems. In addition to the biocorona, cellular membrane‐like structures of sizes ≈1200 nm are adsorbed onto the BIONS (Figure 6C). These residual structures might impact the overall adsorption results even though by definition they are not necessarily considered part of the biocorona. The effect of the cell disruption on the availability of the biomolecules as single molecules is not evaluated in this study. Bead milling could not completely destroy the entire cell walls, and part of the material held as a structural unit, as observed in the TEM images, and might adsorb with a different behavior than individual molecules. However, we seldom saw cellular membrane parts as shown in Figure 6C (red arrow). They do not seem to be very abundant.

DLS measurements show the large sizes varying from 3700 to 8221 nm formed after the interaction of the different biomolecules with the surface (Figure 6D). Remarkably, salts hinder the agglomeration of the BIONs; therefore, the biocoronas in deionized water are the largest. For the remaining conditions, relating size to larger biocoronas is difficult, as other factors, such as the agglomeration of the BIONs in solution, may play a role. Regarding the zeta potential values, the organic material has the same values as those for the cell lysate (Figure S3, Supporting Information), indicating that the organic material was adsorbed onto the surfaces.

The FTIR spectra were employed to characterize the biomolecular content of the corona over the nanoparticle surface. Figure 6E shows the FTIR of iron oxide nanoparticles after exposure to the microalgae lysate under different conditions. Note that there are differences in the intensity of amide II peak at pH 10.0 with respect to the other conditions, which is attributed to structural changes in the proteins.^[^ ^56^ ^]^ The use of FTIR helps, hence, to identify whether the biomolecules have undergone structural changes and if this condition can still be considered for bioprocessing. Some bands belonging to other molecules, such as nucleic acids or pigments, could overlap in the bands mentioned above, and therefore, FTIR analyses were only used as an indicator to identify the presence of the main biomolecules in the biocorona. Other components from the cell lysate, such as pigments (e.g., chlorophyll), which were not quantified, also yield information about the adsorption behavior of other biomolecules through changes in color of the supernatants (Figure 6F).

## 3 Conclusion

This work shows a systematic analysis of biocorona formation in a complex biological environment. We use individual methods to quantify the composition of the biocorona regarding protein, fatty acid, and sugar contents as a function of pH, temperature, and ionic strength to identify the impact of environmental conditions on the interactions taking place on the biomolecule–nanoparticle surface. Our study reveals that the quantities of the biomolecules adsorbed change significantly depending on the conditions, thereby exposing the circumstances that promote the adsorption of each type of biomolecule. Protein adsorption varies depending on the three parameters analyzed in this study, obtaining higher adsorption capacities in the presence of salts, at high temperatures, and at acidic pHs. Lipid adsorption is temperature and pH dependent, obtaining the highest adsorption capacity at the lowest pH (pH 4.0) and temperature (4 °C) studied. Carbohydrate adsorption mainly depends on the pH, attaining the highest capacities at the lowest pH tested.

Although selective adsorption has been discussed in terms of recognition sites, we suggest that this specificity is a matter of the molecule partitioning between the solid and liquid phases, depending on the solubility in the media, hydration level, the physicochemical characteristics of the solid surface as well as of the molecules in the mixture, and on the initial concentration profiles in the system. The results indicate that by adapting the nanoparticle‐to‐biomass mass ratio, it should be possible to separate proteins and lipids from most of the polysaccharides; the former would accumulate on the biocorona while the latter would remain in solution.

This work offers a methodology to analyze and gain insights into the distribution of biomolecules between the solid and liquid phases. This methodology can help researchers better understand and analyze real biological systems due to the resolution gained through the concentration of the biomass on the solid supports. We encourage increasing the knowledge of complex systems and learning about how cellular diversity can impact the distribution of biomolecules on the bio–nano interface.

Finally, we conclude that from a more in‐depth analysis of the data, some natural candidates to enhance and diminish the adsorption of other biomolecules could be identified. In the future, we would like to combine this information with the study of other cellular systems to see if we can modulate the adsorption of particular bio(macro)molecules through the targeted addition of other selected (bio)molecules.

## 4 Experimental Section

4.1

4.1.1

##### Synthesis and Characterization of Iron Oxide Nanoparticles (BIONs)

For the synthesis, we co‐recipitated Fe^2+^, FeCl_2_(H_2_O)_4_, and Fe^3+^, FeCl_3_(H_2_O)_6_ in an alkaline solution (1.8 m NaOH). The final black iron oxide solid phase was repeatedly washed with deionized water until the final conductivity dropped below 200 μS cm^−1^. We recently published details of the particle synthesis and characterization in Abarca Cabrera et al. (2022).^[^ ^17^ ^]^

##### Cell Cultivation and Storage

The cell biomass of microalgae *Microchloropsis salina* (SAG 40.85) was provided by the Chair of Biochemical Engineering (TU München), which obtained the strain from the Culture Collection of Algae at the University of Göttingen, Germany. For maintenance of the strain, modified ASW medium (see Table S1, Supporting Information, for composition) in a sterile shaking flask was used at room temperature and laboratory light.^[41b]^ The cultivation was performed in open thin‐layer cascade photobioreactors using ASW as media.^[^ ^18^ ^]^ The cells were stored at 4 °C in the dark at a concentration of ≈10 g L^−1^ until further use.

##### Determination of Cell Dry Biomass

The algal suspension (2 mL) was centrifuged at 8000 g for 2 min generating a supernatant and a pellet. The supernatant was discarded and replaced with 2 mL of deionized water. This washing step was performed twice to remove the salts from the pellet, which was then dried overnight at 60 °C. The dry biomass was calculated by subtracting the weight of the empty tube from the remaining final and constant mass in the tube.

##### Disruption of M. Salina

The cell disruption was conducted by bead milling using glass beads from 0.25 to 0.5 mm (Carl Roth GmbH + Co. KG, Germany) and biomass in a ratio 1:1 in a mixer mill MM 400 (Retsch, Germany) for 15 min with a frequency of 25 s^−1^.

##### Batch Adsorption of M. Salina Components onto BIONs

The adsorption of the different types of molecules was evaluated by mixing a 1:1 ratio of microalgal biomass and nanoparticles (Equation S1 and S2, Supporting Information). The BIONs in a concentration of 29 g L^−1^ in deionized water were previously sonicated using the ultrasonic homogenizer Branson Digital Sonifier 450 (Emerson Electric Co., USA) for 3 min, at 20% amplitude, 10 s on and 15 s off, in order to decrease the effect of agglomeration due to storage. After disrupting the cells, the lysate and BIONs were mixed in technical triplicates in a 2 mL Eppendorf tube for 1 h at 25 °C and 1000 rpm. After the incubation, an external magnet was employed to separate the supernatant from the BIONs. The BIONs exposed to the lysate were washed twice and resuspended in water for a final concentration of 1 g L^−1^ for size distribution (DLS), zeta potential, and FTIR measurements and stored in a nitrogen atmosphere at 4 °C. The supernatants were stored for further analysis.

##### Quantification of Total Proteins

The BCA assay was employed using the Pierce BCA Protein Assay Kit (Thermo Fisher Scientific Inc., USA), according to the manufacturer's user guide for the analysis of lysate and supernatants. In brief, 200 μL of working reagent were mixed with 25 μL of sample or standard in a Nunc 96‐well microplate (Thermo Fisher Scientific, U.S.A) and the microplate was incubated for 30 min at 37 °C. The absorbance at 562 nm was measured in technical triplicates in an Infinite M200 Microplate Reader (Tecan Deutschland, Germany). The mass balance was verified measuring also the protein loading on the BIONs (1 g L^−1^) with a modified BCA assay which required the separation of the BIONs by centrifugation at 4000 rpm for 5 min after the incubation time using 96‐well filter plates (Pall Corporation, 0.2 μm) with a Nunc 96‐well microplate at the bottom to recover the liquid. BSA was used as standard.

##### Characterization of Proteins by SDS‐PAGE

The adsorbed proteins were identified using SDS‐PAGE with 12% polyacrylamide as the separating gel and 5% as the collecting gel. A 15‐μL solution of sample (lysate, supernatant, or BIONs) was mixed with 15 μL of loading buffer (50 m_M_ Tris, 2% w/v SDS, 10% glycerin, 0.1% w/v bromophenol blue and 100 mm DTT). The gels were run at 180 V, 110 mA, and 30 W for ≈70 min using 10 μL of each sample and the protein standard P7719S (New England Biolabs, U.S.A) and dyed with Coomassie Brilliant Blue R 250 (Carl Roth GmbH + Co. KG, Germany) for 1 h in ethanol/acetic acid. The gels were scanned with the Amersham Typhoon NIR Plus (GE Healthcare Europe GmbH, Germany).

##### Extraction and Direct Transesterification of Fatty Acids

The fatty acid composition was determined by performing a modified direct transesterification as proposed by Griffiths et al. (2010),^[^ ^57^ ^]^ followed by GC. In brief, dried samples were mixed with 900 μL cooled toluene in glass tubes with screw‐cap lids. One hundred microliters of C17‐TAG (4 g L^−1^) were then added as an internal standard. Subsequently, 2,2‐dimethoxypropane (200 μL) and then 2 mL of cooled sodium methoxide were added. The samples were incubated at 80 °C for 20 min at 600 rpm and afterward cooled on ice for 5 min. Two milliliters of a cooled solution of 5% v/v HCl in methanol was added, and the mixture was incubated in the same conditions previously described. Finally, 800 μL of deionized H_2_O and 800 μL of cooled hexane were added. The samples were vortexed and centrifuged for 2 min at 3000 rpm. The upper layer was transferred to a glass vial with an insert and immediately stored at −20 °C for later analysis in GC. Before the transesterification, the nanoparticle content was extracted using the Folch method to collect all lipids attached to the surface.^[^ ^58^ ^]^ The BIONs were resuspended in 0.75 mL of deionized water and mixed with chloroform and methanol using a ratio of 8:4:3 chloroform:methanol:water. The mixture was vortexed for 5 min and then centrifuged for 2 min at 1500 g. The bottom phase containing the extracted lipids was recovered with a syringe and transferred into a glass tube, which was placed under a hood until the chloroform evaporated.

##### Quantification of Fatty Acids

The FAME extract (1 μL) was injected at 280 °C and a split ratio of 25:1 into an Agilent 7890B‐GC equipped with a flame ionization detector and a Stabilwax column (30 m × 0.25 mm ID × 0.25 μm) (Restek GmbH, Germany). The column temperature was increased from 160 °C to 250 °C in increments of 2.5 °C min^−1^ with the detector temperature at 280 °C. Nitrogen at 0.63 mL min^−1^ was used as the carrier gas. The calibration curve was derived from dilutions of a stock solution of C17‐TAG (4 g L^−1^), which were treated with the same procedure as the samples. Marine Oil FAME Mix (Restek GmbH, Germany) was used to identify the retention time of each fatty acid.

##### Quantification of Total Carbohydrates

For total carbohydrates, a colorimetric reaction with phenol–sulfuric acid assay was conducted with a sample pretreatment using acidic hydrolysis.^[^ ^59^ ^]^ First, the samples, after adsorption, were put in glass tubes at 60 °C until dry. The hydrolysis was performed according to Alavijeh et al. (2020).^[^ ^34^ ^]^ In this method, 250 μL of 72% (w/w) sulfuric acid was added to each dried sample, and the samples were then mixed at 30 °C for 1 h. Afterward, 7 mL of deionized water was added, and the tubes were autoclaved at 121 °C for 1 h. After cooling down, an aliquot of 2 mL of each sample was neutralized at a pH between 6 and 8 using calcium carbonate. The samples were centrifuged at 4000 rpm for 10 m in and filtered using 0.2 μL syringe filters for further analysis. For the phenol‐sulfuric acid assay, a 20 μL neutralized sample was mixed with 180 μL of cold and freshly prepared phenol‐sulfuric acid solution containing 4.5 mL phenol (Merck KGaA, Germany) solution at 5% w/v in deionized water and 22.5 mL of sulfuric acid and incubated for 5 m in at 900 rpm and room temperature using microplates. Finally, the microplate was incubated for 35 min at 80 °C. The total sugars were measured at 480 nm, and glucose (Carl Roth GmbH + Co. KG, Germany) was used as the standard.

##### Analysis of Monomeric Compositions

The composition of the hydrolysates was performed as previously reported.^[^ ^60^ ^]^ In brief, an aliquot of 25 μL of the neutralized hydrolysate was transferred to a 96‐well‐polymerase chain reaction (PCR) microplate (Brand 781 350, Germany). The neutralization of the sample was checked by adding 12.5 μL phenol red indicator (0.05 g phenol red in 5 mL 20% ethanol). The samples were then derivatized with the procedure described by Rühmann et al. (2016).^[^ ^59^ ^]^ The samples were measured via liquid chromatography coupled with ultraviolet and electrospray ionization ion trap detection (HPLC–UV–ESI–MS), as previously described.^[^ ^60^ ^]^

##### Surface Characterization after Adsorption

The hydrodynamic diameter and ζ potential of 1 g L^−1^ suspensions of lysates at different pHs and BIONs exposed to the lysate (1 g L^−1^) were measured by DLS and zeta potential using the Zetasizer Ultra (Malvern Panalytical, UK) in a 10 mm cuvette at 25 °C. Each measurement resulted from technical triplicates. The FTIR spectra were acquired using a Bruker ALPHA II spectrometer, measuring 24 scans per sample. BIONs (1 g L^−1^) were precipitated in the diamond crystal from 4000 to 400 cm^−1^. For all measurements, a concave rubber band baseline correction was applied using the software OPUS 8.1. TEM was used to determine the morphology of the BIONs after exposure to the lysate. A 10 μL sample with a concentration of 0.01 g L^−1^ was deposited onto a glow‐discharged carbon‐coated copper grid (Science Services, Germany) until dry. Images were recorded with a Tecnai G2 Spirit (Thermo Fisher Scientific/FEI, NL) transmission electron microscope and processed using ImageJ software v1.53e.

##### Statistical Analysis

The data were represented as mean of three independent adsorption experiments repeated under the same conditions with error bars that depict the minimum and maximum values to illustrate the dispersion of the data set of the adsorption capacities of the different types of biomolecules. Calibration curves using linear regression with correlation coefficients greater than 0.99 were used to calculate the total protein, fatty acids, and total carbohydrates in the samples. The blank, which corresponds to the zero value in the calibration curve, was substracted to the samples to remove the background influence. In the FTIR spectra, the absorbance from 900 to 1200 cm^−1^ was normalized with the peak at 1052 cm^−1^, from 1200 to 2700 cm^−1^ with the peak at 1651 cm^−1^, and from 2700 to 3000 cm^−1^ with the peak at 2922 cm^−1^.

## Conflict of Interest

The authors declare no conflict of interest.

## Author Contributions

L.A.‐C. took care of methodology, validation, formal analysis, investigation, writing the original draft, data curation, visualization, and funding acquisition; O.M. took care of methodology, data curation, validation, formal analysis, investigation, and visualization; V.H. took care of methodology, validation, formal analysis, investigation, and visualization; B.R. took care of methodology, validation, formal analysis, investigation, and writing; J.K. took care of methodology, validation, and formal analysis; M.K. took care of formal analysis and editing; H.D. took care of resources and editing; V.S. took care of resources and editing; S.B. took care of resources, writing the review and editing, supervision, and project administration; P.F‐G. took care of investigation, validation, conceptualization, methodology, writing the review and editing, supervision, and project administration.

## Supporting information

Supplementary Material

## Data Availability

The data that support the findings of this study are available from the corresponding author upon reasonable request.
