# Supplementary material for: Biocorona on Iron Oxide Nanoparticles in a Complex Biotechnological Environment: Analysis of Proteins, Lipids, and Carbohydrates
Source: Small Sci. 2023 Jul 9;3(9):2300064. doi: 10.1002/smsc.202300064 (PMC11935842; doi:10.1002/smsc.202300064)
Supplement: Supplementary file 1 — Supplementary Material [file SMSC-3-2300064-s001.pdf]

Supporting Information

Biocorona on iron oxide nanoparticles in a complex biotechnological environment: analysis of proteins, lipids, and carbohydrates

Lucía Abarca-Cabrera, Olga Milinovic, Viktoria Heitler, Broder Rühmann, Jürgen Kudermann, Massimo Kube, Hendrik Dietz, Volker Sieber, Sonja Berensmeier, Paula Fraga-García*

L. Abarca-Cabrera, O. Milinovic, V. Heitler, S. Berensmeier, P. Fraga-García
Technical University of Munich (TUM), School of Engineering and Design, Department of Energy and Process Engineering, Bioseparation Engineering Group, Boltzmannstraße 15, Garching, 85748, Germany
E-mail: p.fraga@tum.de

B. Rühmann, V. Sieber
Technical University of Munich (TUM) Campus Straubing, Chemistry of Biogenic Resources, Schulgasse 16, Straubing, 94315, Germany

J. Kudermann
Technical University of Munich (TUM), Catalysis Research Center (CRC)
Ernst-Otto-Fischer-Straße 1, Garching, 85748, Germany

M. Kube, H. Dietz
Technical University of Munich (TUM), Department of Biosciences, School of Natural Sciences, Am Coulombwall 4a, 85748 Garching, Germany

Technical University of Munich (TUM), Munich Institute of Biomedical Engineering, Boltzmannstraße 11, 85748 Garching, Germany

Table S1. Composition of ASW media and micronutrients solution

Chemical	Concentration
NaCl	27 g L^{-1}
$MgSO_4$ • 7 H_2O	6.6 g L^{-1}
$CaCl_2$ • 2 H_2O	1.5 g L^{-1}
KNO_3	1 g L^{-1}
KH_2PO_4	0.07 g L^{-1}
$FeCl_3$ • 6 H_2O	0.014 g L^{-1}
Na_2EDTA • 2 H_2O	0.021 g L^{-1}
Micronutrient solution	(1 mL L^{-1})
Micronutrient solution	
$ZnCl_2$	0.04 g L^{-1}
H_3BO_3	0.6 g L^{-1}
$CoCl_2$ •2 H_2O	0.04 g L^{-1}
$MnCl_2$ • 4 H_2O	0.629 g L^{-1}
$(NH_4)_6Mo_7O_{24}$ • 4 H_2O	0.37 g L^{-1}

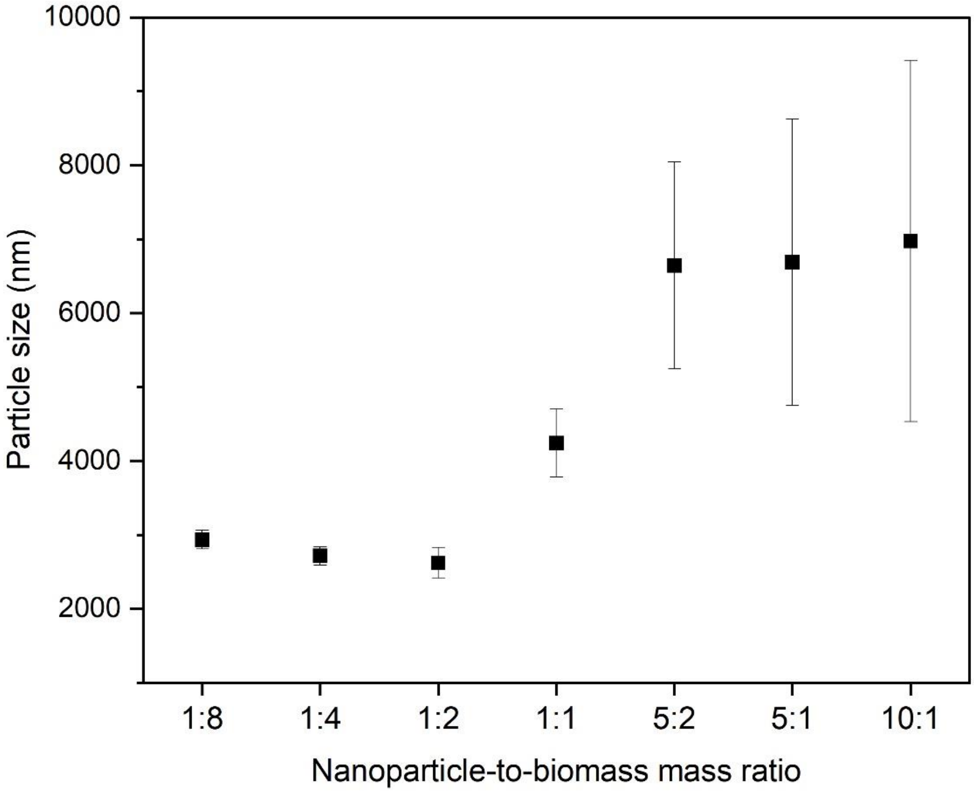

Figure S1. Hydrodynamic diameter of the suspended particles (i.e., BIONs covered with adsorbed biomass) studied at the different BION:lysate mass ratios in Figure 2, at a concentration of 1 g L^{-1} using technical triplicates.

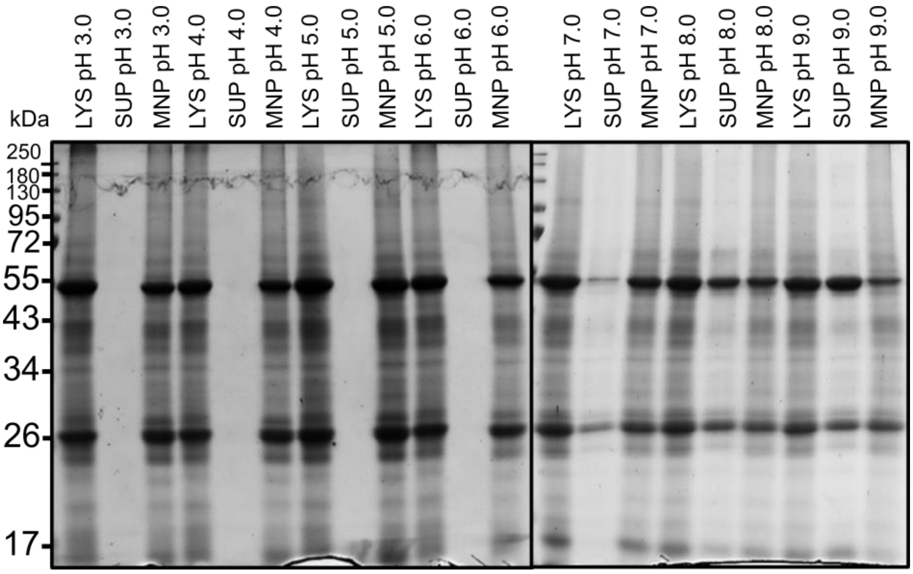

Figure S2. Adsorption of proteins from microalgal lysates onto bare iron oxide nanoparticles at different pHs in deionized water.

Table S2. pH and conductivities of the different conditions applied during the interaction of the biomolecules from *M. salina* and BIONs

Condition	Sample	Conductivity (mS cm^{-1})	pH
	Lysate	41.5 ± 0.93	8.0 ± 0.05
ASW	After contact	38.7 ± 0.90	7.8 ± 0.10
	Supernatant	42.3 ± 0.06	7.7 ± 0.02
	Lysate	0.400 ± 0.01	8.4 ± 0.03
H_2O	After contact	0.473 ± 0.01	8.2 ± 0.05
	Supernatant	0.485 ± 0.01	8.3 ± 0.02
	Lysate	41.5 ± 0.93	8.0 ± 0.05
4°C	After contact	39.2 ± 0.70	7.5 ± 0.03
	Supernatant	42.3 ± 0.10	7.6 ± 0.07
	Lysate	41.5 ± 0.93	8.4 ± 0.03
40°C	After contact	41.3 ± 0.61	7.5 ± 0.02
	Supernatant	40.5 ± 0.10	7.6 ± 0.06
	Lysate	40.0 ± 0.15	4.0 ± 0.03
pH 4.0	After contact	39.4 ± 0.85	4.1 ± 0.11
	Supernatant	41.7 ± 0.40	4.1 ± 0.04
	Lysate	41.0 ± 0.38	6.1 ± 0.05
pH 6.0	After contact	40.8 ± 0.15	6.1 ± 0.07
	Supernatant	41.1 ± 0.06	6.3 ± 0.05
	Lysate	41.2 ± 0.25	8.0 ± 0.09
pH 8.0	After contact	41.5 ± 0.26	7.9 ± 0.12
	Supernatant	41.7 ± 0.31	8.0 ± 0.04
	Lysate	42.4 ± 0.12	10.0 ± 0.06
pH 10.0	After contact	42.3 ± 0.06	9.7 ± 0.54
	Supernatant	41.5 ± 0.06	9.9 ± 0.08

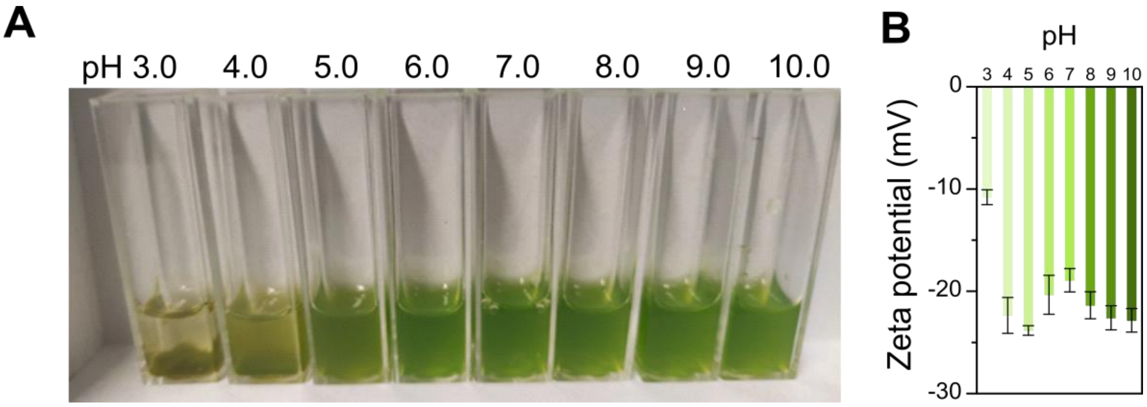

Figure S3. (A) *M. salina* lysates at 1 g L^{-1} adjusted at different pHs. (B) Zeta potential of *M. salina* lysates at 1 g L^{-1} at pHs from 3.0 to 10.0.

Table S3. pKa of the fatty acids studied.

Name of the fatty acid	Chemical name	pKa	Reference
Eicosenoic acid	C20:1	6.49	
Oleic acid	C18:1n9	6.44	
Vaccenic acid	C18:1n7	6.44	
Palmitic acid	C16:0	6.25	[1]
Eicosadienoic acid	C20:2	6.16	
Linoleic acid	C18:2	6.11	
Linolenic acid	C18:3	~6.05	
Palmitoleic acid	C16:1	< 6	

Table S4. Total fatty acid load at different adsorption conditions. Experiments were carried out in technical triplicates. Min and Max values represent the lowest and highest data points in the data set, respectively.

Condition	Load of total fatty acids ($g\ g^{-1}$)	Min value	Max value
ASW	0.039	0.034	0.043
H2O	0.050	0.046	0.052
pH 4.0	0.076	0.068	0.089
pH 6.0	0.056	0.052	0.060
pH 8.0	0.048	0.048	0.048
pH 10.0	0.039	0.035	0.042
4 °C	0.063	0.059	0.065
40 °C	0.033	0.032	0.035

Table S5. Concentration of the most abundant fatty acids found in *M. salina* lysates using technical triplicates in ASW media.

Name	Concentration of fatty acids in lysates (m_M)
C16:0	0.20 ± 0.05
C16:1	0.03 ± 0.01
C18:1n9	0.01 ± 0.00
C18:1n7	0.06 ± 0.01
C18:2	0.09 ± 0.02
C18:3	0.05 ± 0.01
C20:1	0.11 ± 0.03
C20:2	0.10 ± 0.02
TOTAL	0.65 ± 0.15

Calculation of volumes for the batch adsorption of *M. salina* lysates (10 g L^{-1}) and BIONs (29 g L^{-1}) at 1:1 biomass-to-nanoparticle mass ratio. The concentrations are expressed in g L^{-1}.

$$\frac{1}{C_{M.\ salina}}x + \frac{1}{C_{\mathrm{BIONs}}}x = \mathrm{v}_{\mathrm{final}}$$

$$x = \frac{\mathrm{v}_{\mathrm{final}}}{\frac{1}{C_{M.salina}} + \frac{1}{C_{\mathrm{BIONs}}}}$$

Equation S1 $$\mathrm{v}_{M.salina} = x\ \frac{1}{C_{M.salina}}$$

Equation S2 $$\mathrm{v}_{\mathrm{BIONs}} = x\ \frac{1}{C_{\mathrm{BIONs}}}$$

where “C” correspond to concentration in g L^{-1}, “v” to volume, and “x” to mass.

References

1 A.A. Pashkovskaya, M. Vazdar, L. Zimmermann, O. Jovanovic, P. Pohl, E.E. Pohl, *Biophys. J.* **2018**, *114*, 2142.
